# Structure and Chromosomal Organization of Yeast Genes Regulated by Topoisomerase II

**DOI:** 10.3390/ijms19010134

**Published:** 2018-01-03

**Authors:** Ricky S. Joshi, Christoforos Nikolaou, Joaquim Roca

**Affiliations:** 1 Molecular Biology Institute of Barcelona, CSIC, Baldiri Reixac 10, 08028 Barcelona, Spain; rjobmc@ibmb.csic.es; 2 Computational Genomics Group, Department of Biology, University of Crete, 70013 Herakleion, Greece; cnikolaou@biology.uoc.gr

**Keywords:** top1, top2, DNA transcription, epigenetics, gene promoter, chromatin structure, *S. cerevisiae*

## Abstract

Cellular DNA topoisomerases (topo I and topo II) are highly conserved enzymes that regulate the topology of DNA during normal genome transactions, such as DNA transcription and replication. In budding yeast, topo I is dispensable whereas topo II is essential, suggesting fundamental and exclusive roles for topo II, which might include the functions of the topo IIa and topo IIb isoforms found in mammalian cells. In this review, we discuss major findings of the structure and chromosomal organization of genes regulated by topo II in budding yeast. Experimental data was derived from short (10 min) and long term (120 min) responses to topo II inactivation in top-2 ts mutants. First, we discuss how short term responses reveal a subset of yeast genes that are regulated by topo II depending on their promoter architecture. These short term responses also uncovered topo II regulation of transcription across multi-gene clusters, plausibly by common DNA topology management. Finally, we examine the effects of deactivated topo II on the elongation of RNA transcripts. Each study provides an insight into the particular chromatin structure that interacts with the activity of topo II. These findings are of notable clinical interest as numerous anti-cancer therapies interfere with topo II activity.

## 1. Introduction

In all eukaryotic cells, the enzymes termed DNA topoisomerases (topo I and topo II) reduce the topological problems of DNA by producing transient breaks on the DNA strands [1,2]. Topoisomerases are of major clinical interest because they are targets of anti-cancer drugs [3]. Topo I relaxes torsional stress of DNA by cleaving one strand and allowing the rotation of the duplex around the other strand [4]. In contrast, topo II eliminates DNA supercoils and catenates by passing one segment of duplex DNA through a transient double-strand break in another [5,6]. Topo II is essential for unlinking of newly replicated DNA molecules and permits thereby chromosome condensation and segregation [7,8]. Topo I and topo II are required to relax DNA positive and negative supercoils produced by the movement of DNA and RNA polymerases [9]. In this respect, the mechanism of topo II is more efficient than the mechanism of topo I in relaxing chromatinized DNA [10,11]. However, topoisomerase activity is not limited to these fundamental roles. There is overwhelming evidence that topoisomerases have a prominent role in gene expression. At gene promoters, negative supercoiling facilitates the formation of the transcription complex [12,13], where positive supercoiling has the exact opposite effect on transcription initiation [14,15]. In *Saccharomyces cerevisae* (SacCer), Topo I and topo II mutually regulate the topology of DNA to maintain gene promoters in a competent state [16]. Both enzymes facilitate recruitment of RNA polymerase II to promoters [17]. In higher eukaryotes, topo I [18] and topo II [19,20] have been found to interact with RNA polymerase complexes during transcriptional activation and elongation. In yeast cells, topo I and topo II were found to bind mainly to intergenic regions of active genes [17,21]. However, despite these apparently redundant roles, topoisomerases I and II could be implicated differently in the regulation of specific steps of gene expression at different stages. Topo II interacts preferentially with particular gene promoter regions in yeast [21], whereas topo I regulates transcription initiation in other subsets of yeast genes [22] by facilitating nucleosome eviction at gene promoters [23]. Yet, ascertaining where cellular topoisomerases play specific functions is difficult. A clearer evidence of topoisomerase specific roles is found in mammals, which have two isoenzymes of topo II (topo IIa and topo IIb). Topo IIa is essential for chromosome segregation, whereas topo IIb is dispensable for cell division but necessary for normal development [1,7]. Regarding gene transcription, topo IIa and IIb operate at different steps and via distinct mechanisms. For instance, topo IIa facilitates DNA transcription by polymerase II on chromatin templates [19], promotes activation of RNA polymerase I by facilitating the formation of pre-initiation complex [20], functionally interacts with chromatin remodelers [24], and opens chromatin of silent genes during embryogenesis [25]. Topo IIb in turn regulates genes required for neural developmental and is involved in ligand mediated gene expression [26,27,28,29]. Unlike topo IIa, topo IIb produces dsDNA breaks at specific promoter regions to regulate transcription [30,31,32] and interacts with cohesin and CTCF at topological domain borders [33]. Therefore, a key challenge in the field is to elucidate the mechanisms by which cellular topo II activities regulate the transcription of specific gene subsets.

Yeast provides the ideal model system to analyze topoisomerases and gene expression due to its compact, highly gene-dense genome [34]. Despite its small size (12 Mbp), the transcription of this genome is complex, including operon-like transcripts and different genic lengths and intergenic spaces [35]. Transcription directionality, including several bidirectional promoters, is also relevant in gene expression [36]. The interplay between gene position and expression is evident when transcription is regulated within loops formed at gene boundaries [37] and DNA supercoiling throughout the genome [14]. The response to DNA topological stress has been shown to be shaped by specific structural properties of yeast promoters [38].

Here we integrate the findings from three separate studies describing the short term (10 min) and long term (120 min) effects of topo II inactivation on gene transcription. Nikolaou et al. [38] performed a genomic transcription run-on (GRO) in *top2-ts* cells and found a subset of genes that are directly up- and down-regulated by topo II inactivation in SacCer and describe in detail their distinct chromatin profiles. Tsochatzidou et al. [39] leveraged the data produced by Nikolaou et al. to investigate how the response to DNA topological stress extends to affect long genomic regions. Finally, Joshi et al. [11] examined long term (120 min) effects of topo II inactivation and revealed that, when topo II is unable to relieve DNA positive supercoils, there is a stall of Pol II during elongation that causes an abrupt decrease of transcripts of lengths >3 kb.

## 2. Short Term Transcriptomic Changes after Topoisomerase II Inactivation

In order to assess changes in transcription shortly after topo II inactivation in *Saccharomyces cerevisiae* (SacCer), Nikolaou et al. [38] conducted a genomic transcription run-on (GRO) with *TOP2* and thermo-sensitive *top2-ts* strain. These GRO experiments consisted in combining in vivo labeled RNA and array hybridization to calculate the transcription rate (TR) for all genes at a certain time point [40]. Cells were grown at 25 °C and during exponential growth, shifted to 37 °C, incubated for 10 min to inactivate the *top2-ts* enzyme. GRO was subsequently performed by radio-labelling the nascent RNA chains for five minutes. Small fluctuations in gene transcription were observed, 270 genes increased their rate of transcription by >1.5-fold, and 158 genes decreased it by <0.65-fold. Interestingly, these modifications were uncorrelated with RNA abundance changes observed after long inactivation (two hours) of topo II reported in Joshi et al. [11] suggesting an alternative time-dependent mechanism of topo II inactivation stress response. Preliminary filtering revealed 158 topo II sensitive transcripts overlapped with environmental stress response genes [41] and subsequently discarded. In total, 173 upregulated and 97 downregulated transcribed RNAs were deemed to be strictly and directly affected by the inactivation of topo II.

## 3. Analysis of the General Functional Properties of TOP2-Associated Genes

**Figure 1:**
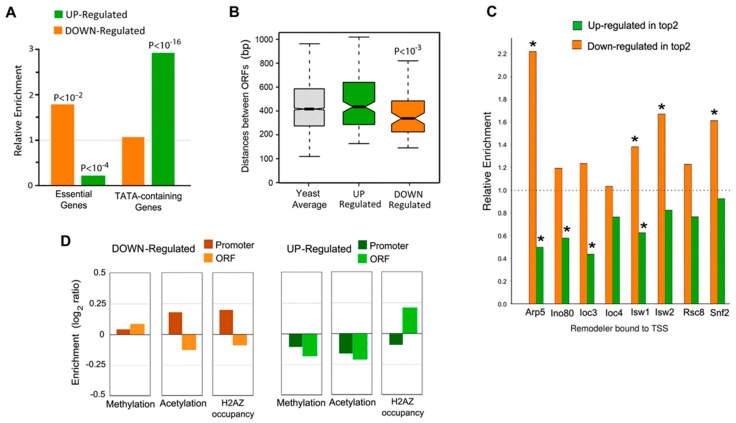
Gene promoter architecture of topo II sensitive transcripts. (**A**) Enrichment of essential and TATA-containing genes calculated as the ratio of observed (up- and down-) over total genes. (**B**) Distance between ORFs, calculated as the distance (bp) from the TSS until the 5′ or 3′ end of the most proximal gene. (**C**) Enrichment of topo II regulated genes among genes occupied by eight chromatin remodelers (**D**) Histone modification enrichments associated to gene subsets downregulated and upregulated after topo II inactivation. Graphs (**A**–**D**) reproduce a summary of the analyses reported by Nikolaou et al. [32]. *: *p* value is less than 0.001.

Initial analyses of the gene subsets regulated by topo II inactivation were compared to two published datasets: 1092 SacCer essential genes [42] and 1073 highly regulated TATA-containing genes [43]. The upregulated subset was poor in essential genes but enriched in TATA-containing genes, whereas the precise opposite trend was observed in the downregulated subset (Figure 1A). Functional enrichment analysis with Gene Ontology (GO) revealed the downregulated genes were related to remodeling of chromatin and transcriptional regulation while the upregulated genes were enriched for membrane transport of polyamines. These differences could be explained by the structural characteristics in the promoter architecture of the two gene sets. In SacCer, gene promoter length is limited by the intergenic distance between ORFs, which on average is about 500 bp [44]. The genes downregulated by topo II deactivation displayed short 5′ intergenic regions whereas the upregulated genes presented intergenic regions longer than the global average (Figure 1B). Interestingly, the occurrence of bidirectional promoters was over-represented in both up and downregulated genes (44% gene average, *p* ≤ 0.002) as compared to the SacCer genome average (26%). Considering these differences in promoter architecture, the occupancy of 126 yeast transcriptional factors [45,46] was evaluated. Although most gene promoters presented a wide profile of transcription factor binding sequences (TFBS) with peak concentration at around position −200 from the transcription start site (TSS), upregulated genes had higher occupancy of TFBS and a concentration peak farther upstream. In turn, downregulated genes accumulated TFBS closer to the TSS, nearby position −150. Regarding the occurrence of individual TFBS, no major differences between up- and downregulated genes were observed. Only some TFBS tend to concentrate downstream position −200 in the downregulated genes (e.g., AF2, OPI1, CAD1, ARO80, PDR1, PAC), while they were allocated farther upstream in the upregulated ones. Other TFBS presented the opposite trends (e.g., MSN2, GAL4, GLN3). Therefore, the transcriptional response to topo II inactivation was dependent on gene promoter type (essential, TATA-containing, TFBS distribution) and size (intergenic distance).

## 4. Chromatin Remodeling and Histone Modification Patterns Associated with TOP2 Deregulated Genes

As discussed in the introduction, mammalian topo II interacts with particular chromatin complexes to regulate gene expression. A study published by Yen et al. [47] described the selectivity of eight chromatin remodeling complexes (Arp5, Isw1, Isw2, Ino80, Loc3, Loc4, Rsc8, Snf2) on nucleosome positioning and organization in SacCer and how in turn, these remodelers shape the transcriptional landscape. Nikolaou et al. assessed the interaction of chromatin remodelers with the TOP2 sensitive gene set. Only Arp5 and Isw1 were both enriched in downregulated genes and depleted in upregulated genes significantly. The chromatin remodelers that were enriched in downregulated genes only were Isw2 and snf2. The chromatin remodelers that were enriched in upregulated genes only were Ino80 and loc3. However, relative to gene average in yeast, the downregulated genes were enriched in all chromatin remodelers assessed, whereas the opposite occurred in the upregulated genes (Figure 1C) suggesting an interplay between chromatin remodelers and topo II in the regulation of gene expression. Finally, the chromatin of the genes deregulated on topo II inactivation were assessed by genome-wide patterns of histone modifications documented for SacCer [48]. The pattern of modifications in downregulated genes was comparable to that found in most SacCer genes (Figure 1D). Only an increase of H2AZK14 acetylation and H3K4 methylation was denoted in promoter and ORF regions, respectively. Upregulated genes presented a quite different landscape (Figure 1D). Histone acetylation and H3K36 di-methylation was reduced in promoters and ORF regions. The occupancy of the histone variant H2AZ was enhanced downstream from the TSS and depleted upstream from it.

The immediate alterations described here are probably not caused by a lack of DNA supercoiling relaxation during transcription elongation as constituent topo I activity is retained and may compensate. Moreover, as topo II inactivation downregulates many essential genes but multiple TATA-harboring gene promoters are upregulated, this suggests that the reported fluctuations occur at the point of transcription activation [49,50]. After the removal of stress-related genes from the topo II sensitive gene set, GO enrichment analysis reported that downregulated genes are enriched for chromatin remodelers and transcription regulators. The upregulated genes were enriched with polyamine transport functions. This was an interesting group of genes as they are rarely seen to be affected in other transcriptome responses. Polyamines regulate chromatin structure by changing DNA conformation and nucleosome stability [51]. Polyamines also change topo II interactions [52,53] and stimulate its activity [54]. Therefore, activating the transport of polyamines may be a homeostatic reaction that tries to enhance topo II activity and stabilize chromatin [38].

**Figure 2:**
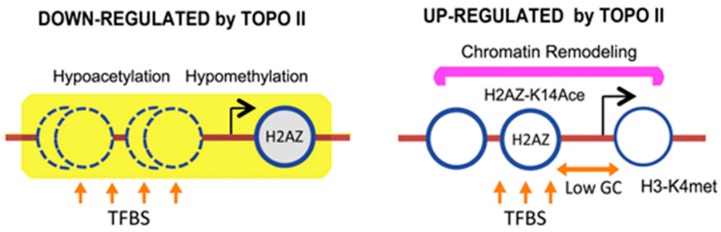
Illustration of the distinctive promoter architecture of yeast genes regulated by topo II. Most significant features of the regulatory regions of SacCer genes positively and negatively regulated by topo II. Adapted from Nikolaou et al. [38].

The chromatin remodeling and histone modification analysis revealed a disparity of promoter architecture between genes affected by topo II inactivation (Figure 2). A possible explanation for the chromatin remodeling change could be due to the presence of nucleosomes relative to the TSS [55]. The enrichment of chromatin remodelers at downregulated genes may be required to displace well-positioned nucleosomes found at −1 and −2. This trait is less important in the upregulated group that has weak peaks of nucleosome position upstream of the TSS. The downregulated group is also enriched in genes with increased marks of transcriptional activation, namely, H3K4 methylation at the ORF and H2AZK14 acetylation at the promoter regions [56,57]. The upregulated genes exhibit a strong bias towards histone marks associated to hypo-acetylation, a heterochromatic hallmark [58,59], and to H3K36 methylation, a repression mark [60,61]. The upregulated genes may thus have chromatin more compacted, which may counteract their fuzzy nucleosome organization and high turnover rate of H2AZ-containing nucleosomes [62,63]. Interestingly, many of the chromatin remodeling and histone modification activities observed here resemble gene sets also regulated by topo II in mammals [24,64,65]. Therefore, fundamental mechanisms of transcription activation and repression mediated by the activity of topo II on gene promoters might be highly conserved in eukaryotic cells, from yeast to humans.

## 5. The Role of Topo II beyond Single Gene Loci

**Figure 3:**
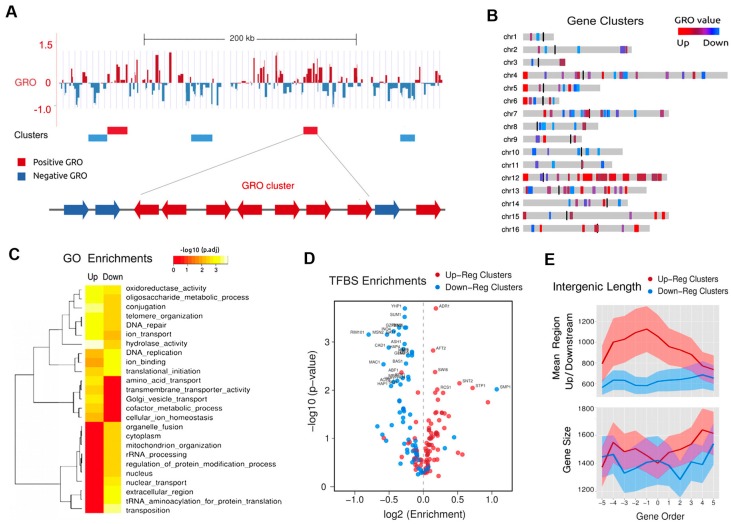
Functional enrichment and regulatory modes of non-random topologically co-regulated clusters. (**A**) Top: example of individual gene genomic transcription run-on (GRO) values and their location in a section of chromosome IV. Red represents increased gene expression (positive) and blue presents decreased (negative). Bottom, cluster definition as a number of contiguous genes with similar GRO values; (**B**) Chromosomal distribution of 116 topologically co-regulated gene clusters (TCGCs). (**C**) GO term enrichment of up and down regulated TCGCs; (**D**) Enrichments of transcription factor binding sequences (TFBS) for 102 yeast transcription factors; (**E**) Top: mean intergenic region length for clusters of 11 consecutive genes. Bottom: same analysis for mean gene length. Shaded bands are 95% confidence intervals. Graphs (**A**–**E**) reproduce a summary of the analyses reported by Tsochatzidou et al. [39].

One of the key characteristics of the SacCer genome is its global gene density as ~70% is made up of coding-genes, many of which exist in tandem. This high genic complexity is compacted into a very small genome of 12 Mb. The controlled response of accumulated topological stress at gene promoters is crucial for transcription [32]. Leveraging GRO data produced by Nikolaou et al. [38], Tsochatzidou et al. [39] assessed the response of extended helical tension to broader genomic regions or gene clusters. Gene clusters were defined as a linear genomic regions where all genes were either upregulated or all downregulated (Figure 3A). Based on permutation analyses of eukaryotic gene order [66], the authors considered significant a cut-off of seven or more contiguous genes. In total, 116 clusters were found. Of these clusters, 50 included exclusively upregulated and 66 entirely downregulated genes with a median number of eight genes for both categories. In total, the clusters comprised 1074 genes representing ~20% of the initial dataset, suggesting that topological stress affects both whole gene clusters as well as single gene promoters.

As inactivation of topo II required cells to be shifted from 25 to 37 °C, for the observed changes in genes expression to be attributed to topological stress and not as a secondary response to heat shock/stress conditions, the authors employed an identical clustering approach in gene expression obtained upon heat shock stress conditions as published in Gasch et al. [41]. Even though a certain degree of clustering was observed, the overlap between the two conditions was insignificant, with limited similarity in the gene expression patterns. Therefore, these results provided evidence that genes allocated within specific chromatin domains were co-regulated by topo II.

## 6. Topologically Co-Regulated Gene Clusters in Linear Chromosomes

The chromosomal distribution of the 116 gene clusters with seven or more genes or topologically co-regulated gene clusters (TCGC) were non-randomly localized throughout the genome (Figure 3A). Downregulated TCGCs were generally found to be located within the vicinity of centromeres or inner regions of chromosomes while upregulated clusters tend to be outward, proximal to the periphery of linear chromosomes. There was also the presence of super-clusters, which were arrays of multiple clusters all up or downregulated by topologically-stressed domains at chromosomes 12, 6 and 7 (Figure 3B). Gene ontology (GO) and gene set enrichment analyses [67] (Figure 3C) showed broad differences between the two types of TCGCs, indicating that their positioning inside the cell nucleus is related to their functional roles. Upregulated gene clusters enriched in peripheral functions are those unrelated to the core molecular processes, as opposed to the downregulated ones that are associated with basic cell functions such as to RNA production, processing and protein synthesis. The three main clusters found were: (i) GO terms enriched in upregulated clusters had functions related to cellular transport, cofactor metabolism and general stress response; (ii) Downregulated GO terms contained basic functions related to RNA transcription, processing and translation; (iii) Functions enriched in both types of clusters include DNA and secondary metabolism. Next, the authors sought to examine the TFBS for both types of TCGCs (Figure 3D). Upregulated clusters presented more complex regulation patterns, with significant enrichments for factors related to chromatin structure and amino acid transport. Downregulated clusters were mostly depleted of TFBS, which can be explained by the enrichment of genes of constitutive expression with less complex regulation. These observations support the observations by Nikolaou et al. [38] where downregulated genes were enriched in essential functions and poor in TATA-boxes. Therefore, topologically co-regulated gene clusters were non-randomly localized throughout the nuclear space and their localization appeared to depend on their promoter architecture and functional relevance.

## 7. Genic Spacing of TCGCs

During transcription, DNA torsional stress accumulates and produces (+) supercoiling in front and (−) supercoiling behind the transcriptional complex [2,9]. Therefore, the size of genes, their intergenic space and the direction of transcription in TCGCs are important for the dissipation of helical stress and concomitant regulation of genome expression. The effect of topo II deactivation is mostly independent of gene size in yeast [16], but is strongly inhibitory in long transcripts (discussed below) therefore correlations of DNA topology with structural genomics are expected TCGCs in TCGCs with similar GRO values. Tsochatzidou et al., observed a high correlation (*p* value ≤ 10^−12^) between the length of intergenic regions and the GRO value of TCGCs, suggesting longer up and downstream regions have a higher capacity to dissipate transcription-induced topological stress. Further analysis of these TCGCs and their GRO values revealed the upregulated clusters and mainly in central genes had long intergenic distances compared to the genome average. Downregulated gene clusters were flanked by much shorter intergenic regions, suggesting that genes with shorter intergenic spacers are more prone to accumulate DNA supercoiling during transcription and be thereby more sensitive to topo II activity. In turn, genes where dissipation of supercoiling can take place throughout long non-transcribed regions are less affected by topo II activity (Figure 3E).

The presence of TCGC in the SacCer genome indicates that not only is the functional role of topological constrains effective locally, as discussed above, but is also determinant across whole gene clusters. The location of these clusters is also relevant to their functional roles as downregulated clusters are primarily found towards the center of the chromosome and include mainly of conserved essential genes. Torsional stress accumulation expectedly shuts down gene expression at sites of high transcription rates such as rRNA transcription where helical tension would build up rapidly upon topo II inactivation. Upregulated clusters, on the other hand, predominantly comprise stress-responsive genes, whose very nature of long intergenic spacers, structural and physical organization and gene transcriptional co-directionality allows these areas of the genome to control DNA supercoiling in order to achieve optimal transcription levels. These features may also extend to gene regulation, function and evolution [36,68,69,70] as the intergenic size has also been found to shape expression levels [71]. Altogether, these observations suggest that chromosomal position and internal organization of TCGC is optimized to interplay with the DNA torsional stress produced during gene transcription.

## 8. Long Term Transcriptomic Changes after Topoisomerase II Inactivation

**Figure 4:**
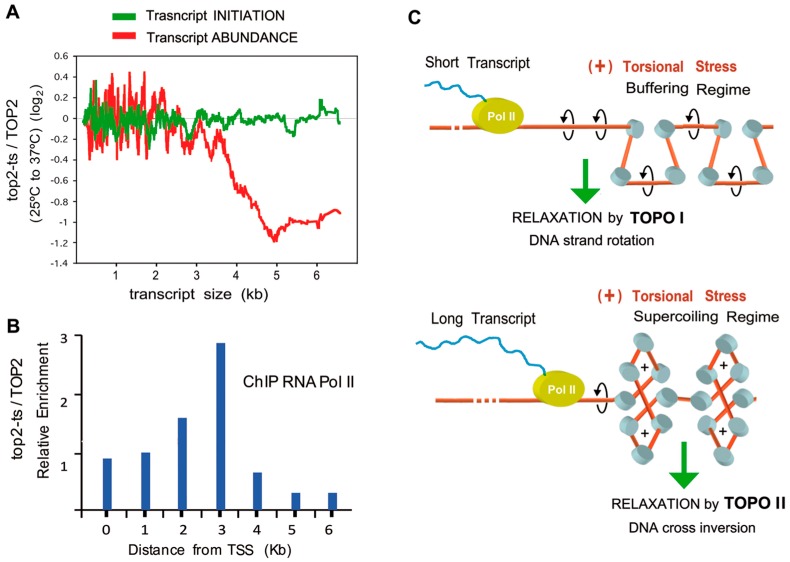
Long term effects of topo II inactivation on transcription. (**A**) Effects of topo II inactivation on transcription according to gene size. Data from GRO experiments TOP2 vs *top2-ts* (green line) and RNA abundance observed by microarrays (red line); (**B**) Intragenic distribution of RNA polymerase II after topo II inactivation. Histograms show the ratio of Pol II density of *top2-ts* relative to control cells; (**C**) Role of topo II during the transcription of long genes. During transcriptional elongation, (+) torsional stress increases in a transcript-length dependent manner. (+) torsional stress diffuses to downstream regions, where it is buffered by chromatin and nucleosome architecture. When this buffering capacity is surpassed, downstream chromatin enters the supercoiling regime. In this chromatin conformation, the DNA strand-rotation mechanism of topo I is not efficient and only the DNA cross-inversion mechanism of topo II is able to remove the (+) DNA supercoils, which would otherwise stall the progression of the RNA polymerase. Graphs (**A**–**C**) reproduce results and the model reported by Joshi et al. [11].

Joshi et al. [11] analyze the effects of long term (120 min) topo II inactivation. Similar to the short term analysis, Joshi et al. grew wild type SacCer and its *top2-ts* derivative at 25 °C until exponential growth. Both the TOP2 and *top2-ts* cells were subsequently shifted to 37 °C during 120 min. Total RNA was extracted and yeast transcriptomic microarrays were used to assess whole transcript changes as a cause of topo II inactivation. Comparison of polyA+ RNA levels revealed that, following topo II inactivation, only 176 transcripts augmented and 195 diminished at significant levels. GO analysis showed 29% of augmented transcripts belonged to networks related to ribonucleoproteins associated with ribosome biogenesis, whereas 39% of the downregulated genes corresponded to categories related to oxidative metabolism. However, though topo II inactivation resulted in minor changes in the yeast transcriptome, a striking observation was that nine percent of the transcriptome representing all transcripts longer than ~3 kb, reduced significantly in expression independently of their functional role (Figure 4A).

## 9. Long Term Inactivation of Topo II Diminishes Long Transcripts

Variation of transcript levels according to the gene size uncovered that average levels do not change for transcripts <2.5 kb. However, an initial reduction appears for all transcripts at ~2.5 kb with a rapid decrease observed at ~3.5 kb (Figure 4A). This reduction of long transcripts is unique to topo II inactivation as the authors performed the same analysis with strains harboring different topoisomerase mutants (Δ*top1* single mutants ([14,22]) and Δ*top1 top2-ts* double mutants ([14,17]). The Δ*top1* single mutant showed no difference in transcript abundance at any length whereas ∆top1 *top2-ts* double mutant displayed overall global reduction of transcription without regard to transcript length relative to controls. This was in agreement with previous studies [13,17,72]. This decrease was not a consequence of transcription initiation as the authors observed no difference in transcript abundance of differing gene lengths in a transcription run-on (GRO) experiment upon topo II inactivation. Therefore, the decrease of long transcripts was attributed to a stall during transcription elongation. The authors subsequently examined the intragenic distribution of Pol II by means of chromatin immunoprecipitation (ChIP). The distribution of Pol II in the *top2-ts* relative to TOP2 cells presented a similar pattern irrespective of transcript size (Figure 4B). Topo II inactivation did not alter Pol II allocation up to 2 kb downstream the TSS. However, Pol II accumulated at distances around 3 kb from the TSS; and was depleted beyond 4 kb from the TSS. These observations corroborated that topo II inactivation does not reduce transcription initiation of long genes, as revealed by the GRO experiments. However, the absence of topo II activity produced an obstacle for the progression of Pol II after transcribing 3 kb.

Previous studies have described the function of DNA topoisomerases in relieving helical stress generated during transcription elongation [1,2]. Failure to do so results in a stall of polymerases as described in vitro [19,73] and an ensuing impairment of transcription in vivo [74]. Hence, this dependence on the length of transcripts is expected in cells with reduced topoisomerase activity, as longer transcripts will accumulate more (+) supercoiling stress. Up to ~3 kb, (+) torsional stress can be either removed by topo I or not accumulated sufficiently to stall the advancement of Pol II. Beyond this length, topo II is essential to avoid stalling of Pol II. This critical length of DNA reveals how intracellular chromatin allows for plasticity to buffer low levels of (+) topological stress. When this buffering power of chromatin is exceeded, the supercoiling regime takes place [75,76,77]. This transition is likely to happen during general transcription, since the generation rate of DNA torsional stress by Pol II is higher than the relaxation rate operated by topoisomerases [76,78]. The abrupt reduction of transcripts >3 kb is likely due to this transition of chromatin structure from the buffering regime to the supercoiling one (Figure 4C). This model is further supported by in vitro studies showing that chromatin is optimally relaxed by topo II when the superhelical density of DNA is >0.04 [10], the point in which chromatin enters the supercoiling regime [75,76,77].

## 10. Conclusions and Perspectives

Topoisomerase II is the only cellular enzyme that alters DNA topology by means of a DNA cross-inversion mechanism. This capacity allows the removal and introduction of supercoils into DNA, as well as catenate–decatenate and knot–unknot DNA molecules. As DNA topology is one of the fundamental determinants of chromosome and chromatin structure and dynamics, it could be anticipated that topo II is implicated at multiple steps of the regulation of gene expression. The studies summarized in this review corroborate these expectations. Topo II seems to regulate numerous genes according to their promoter architecture. Future research might uncover whether this regulation involves DNA topology changes, only DNA cleavage, or only a structural interaction to facilitate or preclude the initiation of transcription. During transcription elongation, topo II DNA relaxation activity emerges also as a regulator of the progression of RNA polymerases. Future studies might assess how this function affects the stability of transcribing factories and other associated processes, such as RNA splicing. Finally, the occurrence of DNA supercoiling waves that can spread beyond a single gene substantiates the authentic notion of DNA topological domains, allowing TCGCs to be co-regulated by topo II. All these regulatory mechanism and pathways were inferred from studies in budding yeast, but are likely to be conserved in all eukaryotic cells. Their better understanding will permit to optimize the anti-cancer therapies that target human topo II activities.

## Supplementary Materials

Supplementary File 1
