# Supplementary figures and images for: Structure and Chromosomal Organization of Yeast Genes Regulated by Topoisomerase II
Source: Int J Mol Sci. 2018 Jan 3;19(1):134. doi: 10.3390/ijms19010134 (PMC5796083; doi:10.3390/ijms19010134)

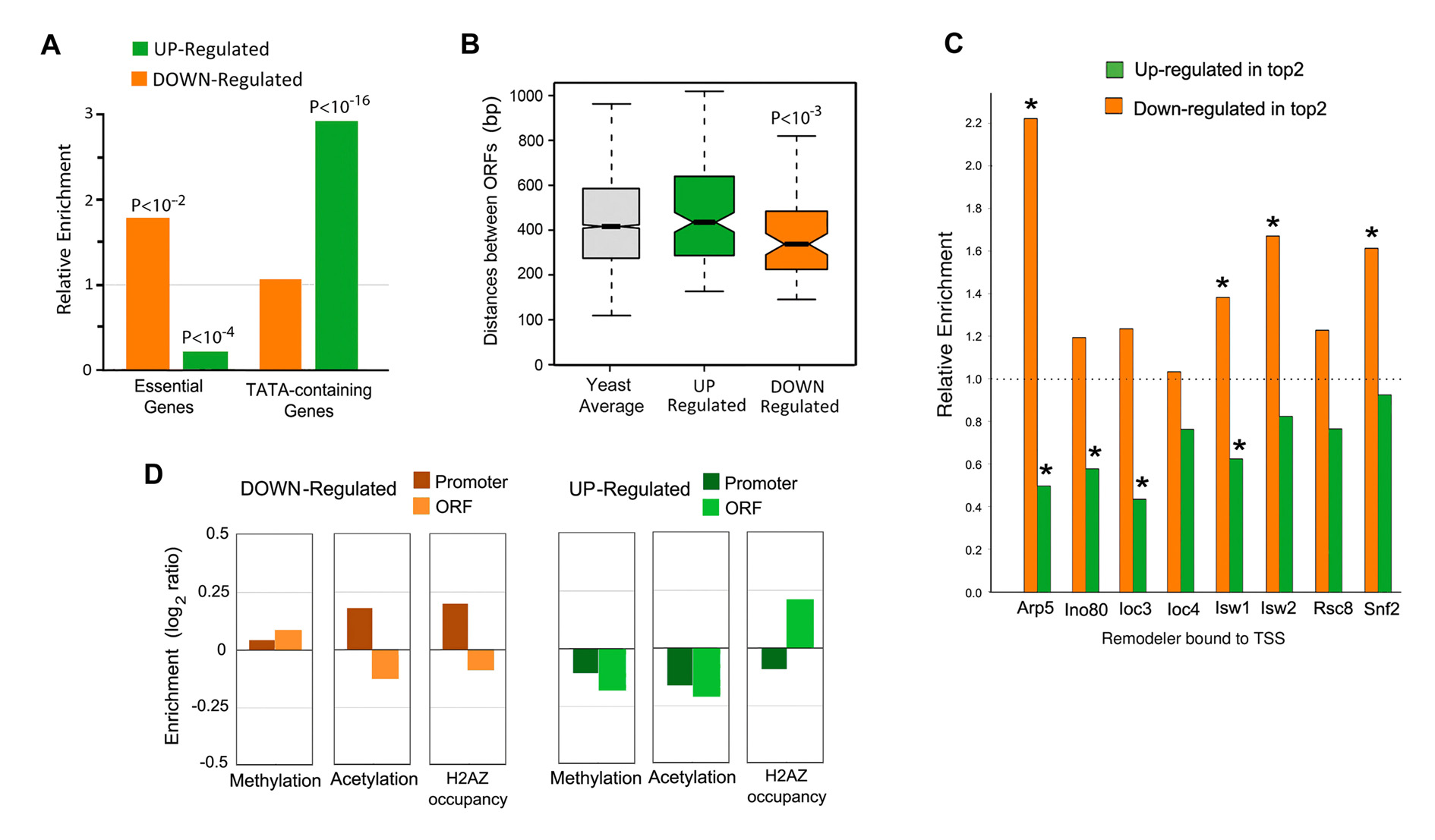

Supplement: Supplementary File 1 [file ijms-19-00134-s001.zip › FIGURES 300/Fig 1.jpg]

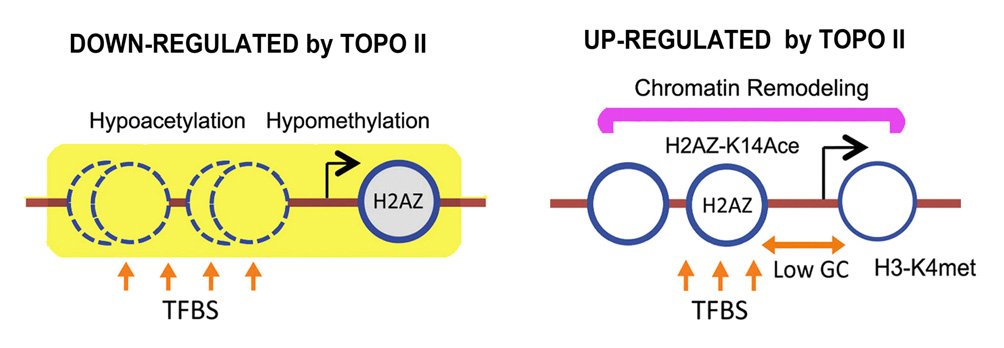

Supplement: Supplementary File 1 [file ijms-19-00134-s001.zip › FIGURES 300/Fig 2 .jpg]

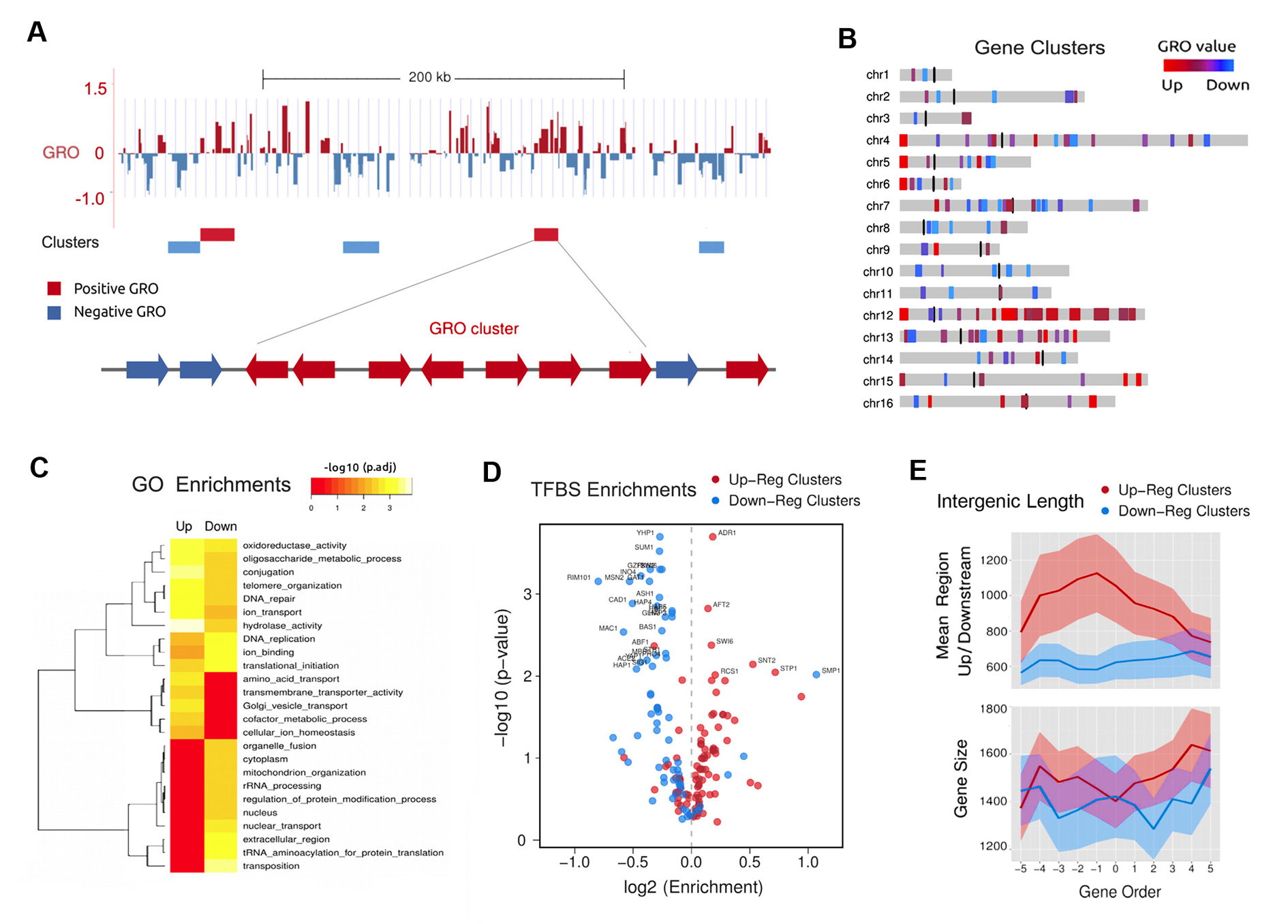

Supplement: Supplementary File 1 [file ijms-19-00134-s001.zip › FIGURES 300/Fig 3 .jpg]

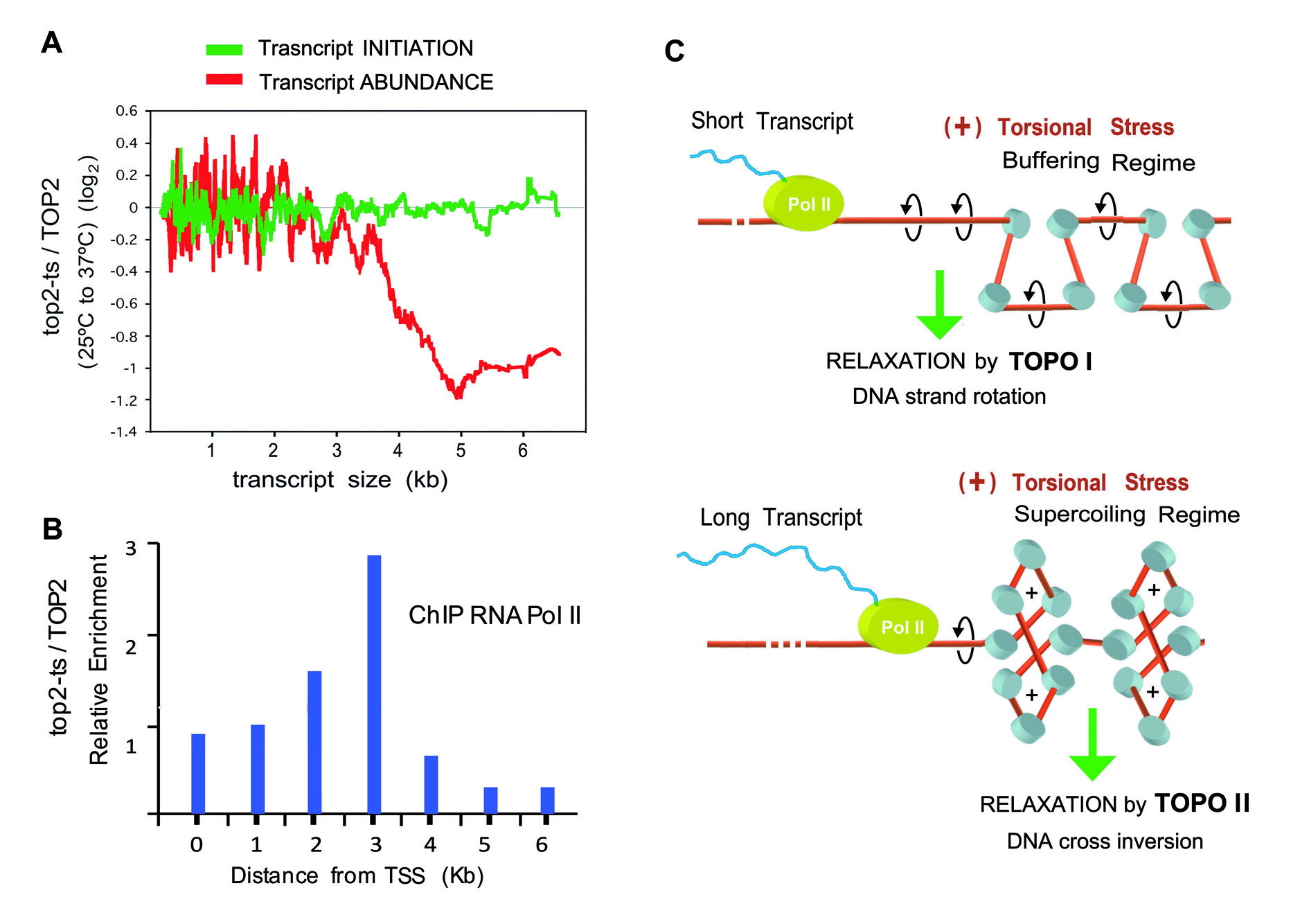

Supplement: Supplementary File 1 [file ijms-19-00134-s001.zip › FIGURES 300/Fig 4 .jpg]
